# Observation of structural and vascular features of retina and choroid in myopia using ultra-widefield SS-OCTA

**DOI:** 10.1186/s12886-024-03473-y

**Published:** 2024-05-07

**Authors:** Yuanyuan Si, Kunpeng Pang, Yanling Song, Xia Zhang, Hongling Yang, Yan Cui

**Affiliations:** 1 Department of Ophthalmology, Qilu Hospital of shandong University, Shandong University, 107 Wenhua Xi Road, Jinan, 250063 Shandong Province China; 2 Cheeloo College of Medicine, Shandong University, Jinan, China; 3 First Clinical Medical College, Shandong University of Traditional Chinese Medicine, Jinan, China

**Keywords:** Retina capillary, Choroid vessels, Myopia, SS-OCTA

## Abstract

**Background:**

To find the relationship between the changes of retinal and choriodal structure/ vascular densities (VD) and the myopia progress.

**Methods:**

126 eyes of 126 age-matched young participants were divided into three groups: Emmetropia and Low Myopia (EaLM) (33 eyes), Moderate Myopia (MM) (39 eyes), and High Myopia (HM) (54 eyes). Fundus images measuring 12 × 12 mm were captured using ultra-widefield swept-source optical coherence tomography angiography (SS-OCTA). Each image was uniformly divided into nine regions: supra-temporal (ST), temporal (T), infra-temporal (IT), superior (S), central macular area (C), inferior (I), supra-nasal (SN), nasal (N), and infra-nasal (IN). Various structural parameters, including inner retina thickness (IRT), outer retina thickness (ORT), and choroid thickness (CT), were assessed, and the VD of the superficial capillary plexus (SCP), deep capillary plexus (DCP), choriocapillaries (CC), and choroid vessels (ChdV) were quantified.

**Results:**

CT in upper fundus exhibited a significant reduction from EaLM to MM. Additionally, ORT (ST, S. SN, C, N, IT, I, IN), CT (ST, S, SN, T, C, N, IT, I, IN) and VDs of SCP (ST, S, C, I, IN), DCP (ST, S, T, C, I) and ChdV (T, N, I, IN) were statistically diminished in EaLM compared to HM. Furthermore, IRT (N), ORT (N, IN), CT (S, SN, T, C, IT, I) and VDs of SCP (I, IN) and DCP (I) exhibited significant decreases as MM progressed towards HM. Intriguingly, there was a notable increase in the VD of CC (ST, S, T, C, N) as myopia progressed from MM to HM.

**Conclusion:**

Significant changes in retinal and choroid structure and vascular density occur as moderate myopia advances to high myopia. Efforts to curb myopia progression to this stage are essential, as the failure to do so may lead to the development of corresponding retinopathy.

**Supplementary Information:**

The online version contains supplementary material available at 10.1186/s12886-024-03473-y.

## Introduction

Myopia represents a burgeoning and noteworthy global public health concern, affecting a significant portion of the world’s population, exceeding 2 billion individuals (28.3% of the global populace). Within this cohort, there are 277 million individuals (4.0%) grappling with the challenges of high myopia (HM) [1]. The progression of myopia is concomitant with a cascade of alterations in the vascular circulation and structural characteristics of the ocular fundus. Metabolic irregularities commonly manifest in the retinal and choroid tissues of HM patients, profoundly influencing the circulatory dynamics of the retina and choroid. Furthermore, mechanical elongation of the ocular axis and the accompanying myopia progression engender a reduction in choroidal and retinal pigment epithelium (RPE) thickness, ultimately leading to a sequence of degenerative lesions within the fundus, resulting in varying degrees of visual impairment in HM individuals [2]. It is noteworthy that the alterations in fundus blood flow mainly stem from changes in choroidal hemodynamics [3]. Recent research by Zhang and colleagues has identified a diminished choroidal blood perfusion as the principal instigator of myopia progression in humans [4]. Reduced choroidal blood flow has been found in eyes with high myopia [5]. A growing view that choroid plays a pivotal role in ocular growth and the development of refractive anomalies such as myopia [6]. Consequently, it is paramount to elucidate the changes of microstructural and vessel features of myopia fundus, especially choroidal vascular alterations.

Prior investigations have focused on the assessment of the choroid vascular index (CVI) in eyes afflicted with HM employing swept-source optical coherence tomography angiography (SS-OCTA), which provides insight into the cross-sectional choroidal blood flow at specific fundus locations [7]. Nevertheless, few studies have directly quantified the vascular density (VD) of choroid vessels (ChdV) within the HM fundus region. Recently, two studies have measured ChdV VD in sub-regions of the peripheral fundus, utilizing dimensions of 20 × 24 mm and 17 × 17 mm [3, 8]. Quantitative assessment of choroidal vasculature by SS-OCTA technologyis still in its exploratory stage currently [9]. To date, there has been a dearth of investigations evaluating ChdV VD in the central and mid-peripheral regions within a 12 × 12 mm field and its association with AL and spherical equivalent (SE) in HM eyes. The advanced application of SS-OCTA will empower us to scrutinize the fundus’s structural and vessels variations during myopia progression, with particular emphasis on ChdV. Moreover, this approach will enable us to unveil the relationships between these changes and axial length, as well as spherical equivalent.

## Materials and methods

### Study participants

Clinical data from 126 eyes of 126 eligible participants were gathered between Oct 1, 2023 and Feb 1, 2024 at the Department of Ophthalmology, Qilu Hospital (Shandong University, Shandong, China). All individuals underwent a comprehensive ophthalmic assessment, including evaluations of best corrected visual acuity (BCVA), refraction, intraocular pressure (IOP), AL, visual field assessment, dilated fundus examinations, and ultra-wide field SS-OCTA. Subjects without other eye disorders except for refraction errors and BCVA above 1.0 were included in the study. The eyes were categorised into three groups based on SE: Emmetropia and Low Myopia (EaLM) group (− 3.00D ≤ SE ≤ + 0.75D, *n* = 33), Moderate Myopia (MM) group (− 6.00D < SE ≤ − 3.00D, *n* = 39), and HM group (SE ≤ − 6.00D or AL ≥ 26.5 mm, *n* = 54). Grouping criterion reference《Expert Consensus on Prevention and Control of High Myopia (2023)》.

The inclusion criteria were (1) age of 18–40 years; (2) BCVA ≥ 1.0; and (3) 10mmHg ≤ IOP ≤ 21 mmHg (1 mmHg = 0.133 kPa); (4) 21.5 mm < AL < 29.00 mm. Exclusion criteria were as follows: (1) non-compliance with the inclusion criteria; (2) a history of neurological or systemic conditions, such as hypertension and diabetes; (3) prior intraocular or refractive surgery; (4) a history of ocular trauma; (5) presence of media opacity; (6) medication usage within the preceding 2 weeks; and (7) ocular inflammation or retinal disorders, including retinoschisis or retinal holes.

This study was conducted in strict adherence to the ethical principles outlined in the Declaration of Helsinki, and it received approval from the Ethics Committee of Qilu Hospital, Shandong University, with informed consent being obtained from all participants.

### Ultra-widefield SS-OCTA image acquisition

**Fig. 1:**
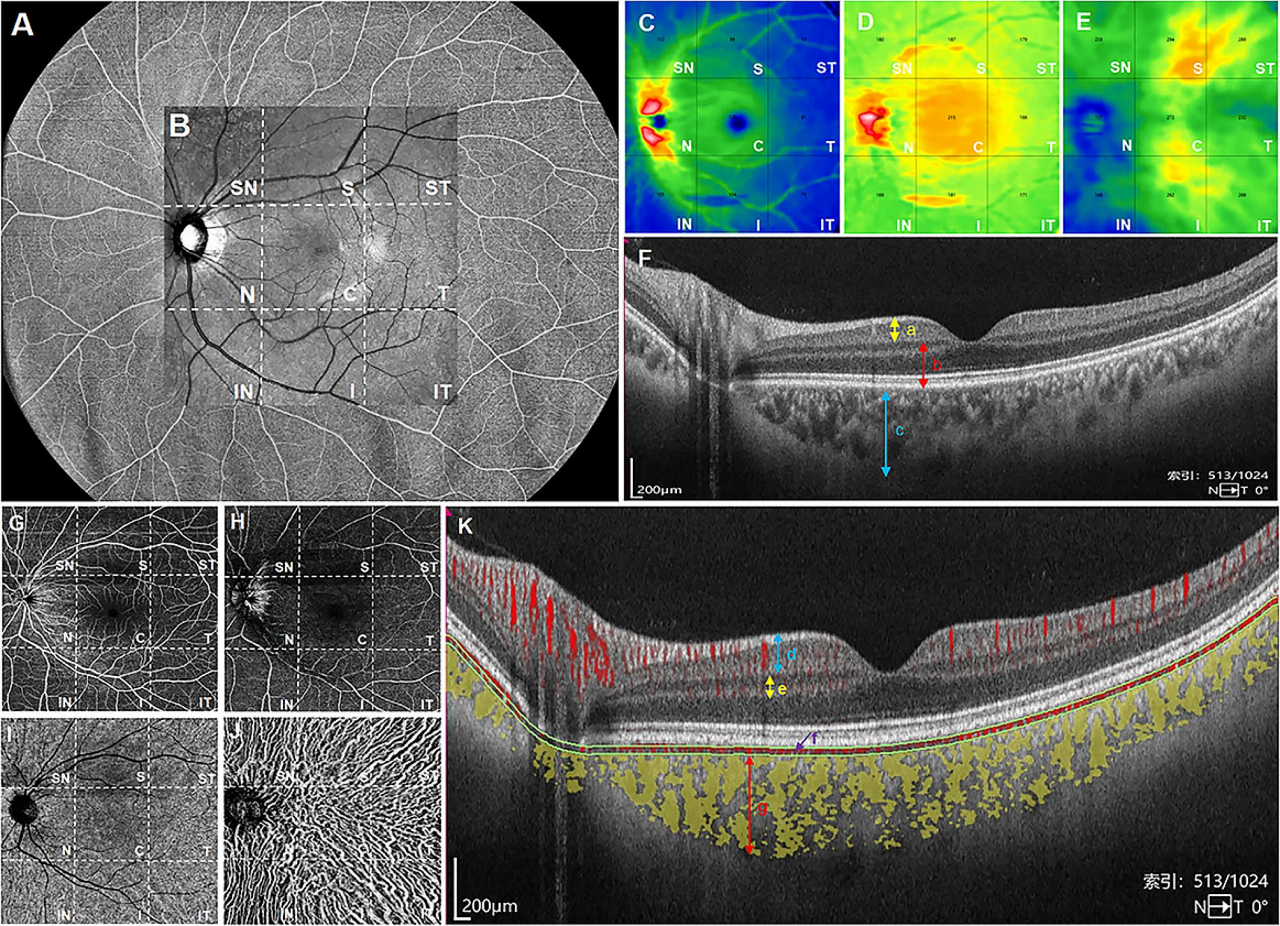
Quantification of thickness and VD in each fundus layer using ultra-wide field OCTA. Scanned with the maximum range of 24 × 20 mm using ultra-wide field OCTA **(A)**. For this study, fundus image data within a 12 × 12 mm area was chosen for quantitative analysis, and automatically segmented into nine 4 × 4 mm regions (ST, S, SN, T, C, N, IT, I and IN) **(B)**. Each cross-sectional image was automatically processed to create topographic maps for three layers, namely, IRL, ORL and choroid layer (**C**–**E**), and four-layer binary vascular images, specifically, SCP, DCP, CC and ChdV (**G**–**J**), all achieved through the built-in software. **(F)** illustrated thickness imaging tomography at the central fovea level, with the distance between the two-way arrows indicating IRT (**a**, **C**: yellow), ORT (**b**, **D**: red), and CT (**c**, **E**: blue), respectively. **(K)** depicted vascular imaging tomography at the central fovea level, with red and yellow areas representing blood flow signals. The regions within the two-way arrow denote SCP (**d**, **G**: blue), DCP (**e**, **H**: yellow), and ChdV (**g**, **J**: red), while the area within the green line represents CC (**f**, **I**: purple), respectively. VD, vessel density; ST, supra-temporal; S, superior; SN, supra-nasal; T, temporal; C, central macular area; N, nasal; IT, infra-temporal; I, inferior; IN, infra-nasal; IRL, inner retinal layer; ORL, outer retinal layer; IRT, inner retinal thickness; ORT, outer retinal thickness; CT, choroid thickness; SCP, superficial capillary plexus; DCP, deep capillary plexus; CC, choriocapillaris; ChdV, choroid vessels

Patients were subjected to imaging using a 400 kHz ultra-wide field SS-OCTA instrument (BM-400 K BMizar; TowardPi Medical Technology Co., Ltd., Beijing, China) equipped with a scanning laser source. The system possessed a scanning depth of 6 mm, a maximal scanning area of 20 × 24 mm (Fig. 1A), with 400,000 scans per second. It utilizes a swept-source vertical-cavity surface-emitting laser (VCSEL) with a wavelength of 1060 nm, providing a transverse resolution of 10 μm and in-depth resolution (optical) of 3.8 μm in tissue. Notably, the entire scanning procedure was completed in under 15 s. Moreover, the inclusion of an ultrahigh frequency real-time eye-tracking function (128 Hz) and subsequent motion correction processes ensured the mitigation of imaging biases resulting from ocular movement. The software generated 1280 cross-sectional images from the B-scan and performed automatic analysis of fundus VD and thickness within these images utilising a high-order moment amplitude decorrelation angiography algorithm [10]. VD was defined as the percentage of vascularized area in relation to the total area of the montage image at a specific vascular layer.

Each eye underwent dual scans, and the resultant data was averaged. Any images of subpar quality, characterized by pronounced motion artifacts or substantial segmentation errors, were excluded and subsequently retaken. Furthermore, all OCTA assessments were conducted by a proficient operator. These examinations were scheduled between 8:00 AM and 11:00 AM and were carried out with pupils in their normal state to minimize the potential influences of circadian rhythm and anti-cholinergic medications on the study outcomes.

### Image analysis

The 12 × 12 mm B-scan mode was selected, and subsequently, all fundus images were quantified and partitioned into 9 square grids, delineated as supra-temporal (ST), superior (S), supra-nasal (SN), temporal (T), central macular area (C), nasal (N), infra-temporal (IT), inferior (I), and infra-nasal (IN) regions (Fig. 1B). At the same time, each layer of horizontal structure and vascular imaging tomography scanned in this 12 × 12 mm range can be obtained.

This OCTA device with built-in algorithms that can automatically calculate and quantify the thickness and vascular density of each layer of fundus. Leveraging this technology, we were able to monitor the thickness of three structural layers, specifically inner retinal thickness (IRT), outer retinal thickness (ORT), and choroid thickness (CT) (Fig. 1C-E). Figure 1F illustrated thickness imaging tomography at the central fovea level, from which we can see that, IRT corresponds to the distance from the internal limiting membrane (ILM) to the inner plexiform layer (IPL), ORT spans from the IPL to the Bruch’s membrane (BM), and CT encompasses the distance from the BM to the choroid-sclerotic interface (CSI) (Fig. 1F, a-c). Furthermore, the quantification of VD in four vascular layers, including the superficial capillary plexus (SCP), deep capillary plexus (DCP), choriocapillaris (CC), and choroid vessels (ChdV) was executed (Fig. 1G-J). Figure 1K depicted vascular imaging tomography at the central fovea level, from which we can see that, the SCP extends from the ILM to 9 mm above the IPL, the DCP encompasses the region from 9 mm above the IPL interface to 9 mm below the outer plexiform layer (OPL), the CC is located 29 mm below the BM, and the ChdV spans from 29 mm beneath the BM to the CSI (Fig. 1K, d-g).

### Statistical analysis

Continuous variables were tested for normal distribution with Shapiro-Wilk test, after which they were examined by the Levene test to determine the homogeneity of variance. Chi-square test was used for gender comparison among the three groups, and one-way analysis of variance (ANOVA) test was used to realize the comparison of differences between the three groups of data, followed by the Bonferroni post hoc test for pairwise comparisons. Gender is expressed as composition ratio, and other count data results were reported as mean ± SEM (Standard Error of the Mean). Pearson’s correlation analysis was used to estimate correlation among the studied parameters. The tests above were performed with SPSS Statistics Version 26.0 Software package (IBM; Armonk, New York, USA). *P* < 0.05 was considered to be statistically significant.

## Results

### General Information and ocular characteristics

A total of 126 eyes from 126 subjects were included in the study, with 33 eyes in the EaLM group, 39 eyes in the MM group, and 54 eyes in the HM group. The distribution of age and gender did not reveal any significant differences among the groups (Table 1). However, there were significant differences in SE and AL observed among the three groups (*p* = 0.000, Table 1).

**Table 1:** General Information and Ocular Characteristics of three groups

Characteristics	EaLM (M/F)	MM (M/F)	HM (M/F)	*P*
Number (eyes)	*N* = 33 (13/20)	*N* = 39 (11/28)	*N* = 54 (22/32)	
Age (y)	26.67 ± 1.00	24.85 ± 0.75	26.41 ± 0.86	0.316
Gender(M/F, %)	39.4/60.6	28.2/71.8	40.7/59.3	0.428
SE (-D)	1.30 ± 0.19	4.68 ± 0.12	7.63 ± 0.14	0.000**
AL (mm)	23.96 ± 0.18	24.96 ± 0.10	26.62 ± 0.12	0.000**

EaLM, Emmetropia and Low Myopia; MM, Moderate Myopia; HM, High Myopia; M, male; F, female; SE, spherical equivalent; AL, axial length. P: difference between 3 groups by ANOVA, *p*<0.01 is marked by **

### Regional thickness analysis of retina and choroid

**Fig. 2:**
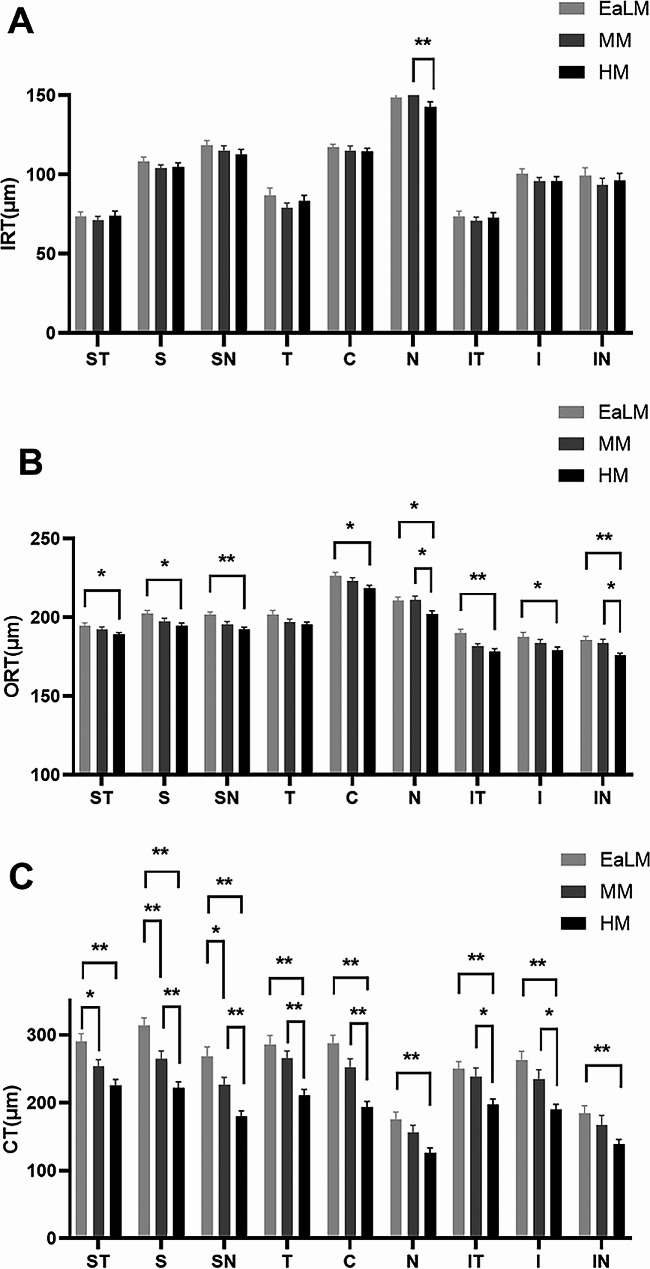
Thickness (µm) of retinal and choroid layers quantified and compared across nine regions for three groups. EaLM, Emmetropia and Low Myopia; MM, Moderate Myopia; HM, High Myopia. IRT, inner retinal thickness; ORT, outer retinal thickness; CT, choroid thickness. ST, supra-temporal **(A)**; S, superior **(B)**; SN, supra-nasal **(C)**; T, temporal **(D)**; C, central macular **(E)**; N, nasal **(F)**; IT, infra-temporal **(G)**; I, inferior **(H)**; IN, and infra-nasal **(I)**. Statistically significant values are shown with */**; *, *p* < 0.05; **, *p* < 0.01

HM exhibited significantly thinner IRT in the N region (*p* = 0.004, Fig. 2A), ORT in the N (*p* = 0.017, Fig. 2B) and IN (*p* = 0.012, Fig. 2B) regions, as well as CT in the S (*p* = 0.009, Fig. 2C), SN (*p* = 0.004, Fig. 2C), T (*p* = 0.000, Fig. 2C), C (*p* = 0.000, Fig. 2C), IT (*p* = 0.013, Fig. 2C) and I regions (*p* = 0.011, Fig. 2C) when compared to the MM group. Additionally, the analysis of thickness revealed that in the MM group, CT displayed significant reductions when compared to the EaLM group in the ST (*p* = 0.048, Fig. 2C), S (*p* = 0.007, Fig. 2C), and SN regions (*p* = 0.026, Fig. 2C). Furthermore, when compared to EaLM, seven regions in the HM group exhibited notably thinner ORT, excluding the T region (ST: *p* = 0.041, S: *p* = 0.020, SN: *p* = 0.001, C: *p* = 0.012, N: *p* = 0.032, IT: *p* = 0.002, I: *p* = 0.043, IN: *p* = 0.002; Fig. 2B). Intriguingly, across all nine 4 × 4 mm regions, CT was significantly decreased in HM in comparison to EaLM (all: *p* < 0.01; Fig. 2C). Additional details can be found in Additional file 1.

Furthermore, the correlation analysis demonstrated that ORT and CT in all nine 4 × 4 mm regions exhibited negative correlations with AL and SE (all: *p* < 0.05; Table 2).

**Table 2:** Correlation of thickness (µm) with AL (mm) and SE (-D) in 9 regions

Region	Layer	AL (mm)		SE (-D)	
		Coefficient	*P*-value	Coefficient	*P*-value
IRT	ST	0.002	0.985	-0.010	0.912
	S	-0.067	0.458	-0.117	0.190
	SN	-0.067	0.454	-0.160	0.075
	T	-0.071	0.430	-0.038	0.673
	C	-0.034	0.709	-0.065	0.469
	*N*	-0.092	0.305	-0.156	0.080
	IT	-0.027	0.770	-0.032	0.725
	I	-0.156	0.082	-0.165	0.065
	IN	-0.018	0.843	-0.062	0.492
ORT	ST	-0.308	0.001**	-0.268	0.003**
	S	-0.246	0.006**	-0.266	0.003**
	SN	-0.303	0.001**	-0.313	0.000**
	T	-0.216	0.015*	-0.218	0.014*
	C	-0.236	0.008**	-0.284	0.001**
	*N*	-0.276	0.002**	-0.292	0.001**
	IT	-0.304	0.001**	-0.272	0.002**
	I	-0.226	0.012*	-0.245	0.006**
	IN	-0.293	0.001**	-0.300	0.001**
CT	ST	-0.232	0.010*	-0.372	0.000**
	S	-0.333	0.000**	-0.459	0.000**
	SN	-0.371	0.000**	-0.418	0.000**
	T	-0.378	0.000**	-0.435	0.000**
	C	-0.420	0.000*	-0.494	0.000**
	*N*	-0.301	0.001**	-0.333	0.000**
	IT	-0.250	0.005**	-0.326	0.000**
	I	-0.332	0.000**	-0.410	0.000**
	IN	-0.269	0.002**	-0.315	0.000**

Statistically significant values are shown with */**, *P*<0.05 is marked by *, *P*<0.01 is marked by **. ST, supra-temporal; T, temporal; IT, infra-temporal; S, superior; C, central macular area; I, inferior; SN, supra-nasal; *N*, nasal; IN, infra-nasal; IRT, inner retinal thickness; ORT, outer retinal thickness; CT, choroid thickness; AL, axial length; SE, spherical equivalent

### Regional VD analysis of retina and choroid

**Fig. 3:**
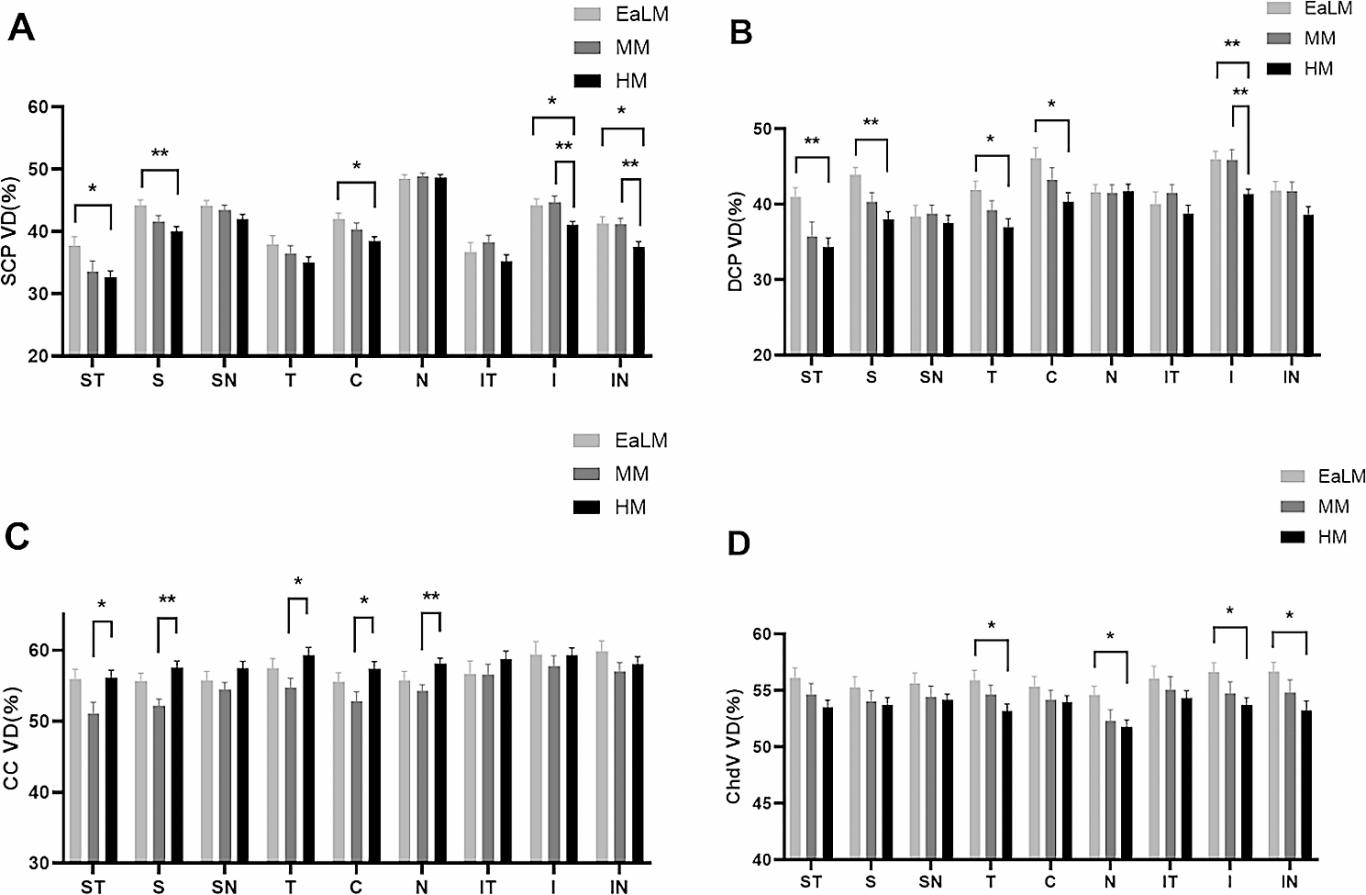
VD (%) of retinal and choroid layers quantified and compared across nine regions for three groups. EaLM, Emmetropia and Low Myopia; MM, Moderate Myopia; HM, High Myopia. VD, vascular density; SCP, superficial capillary plexus; DCP, deep capillary plexus; CC, choriocapillaris; ChdV, choroid vessels. ST, supra-temporal **(A)**; S, superior **(B)**; SN, supra-nasal **(C)**; T, temporal **(D)**; C, central macular **(E)**; N, nasal **(F)**; IT, infra-temporal **(G)**; I, inferior **(H)**; IN, and infra-nasal **(I)**. Statistically significant values are shown with */**; *, *p* < 0.05; **, *p* < 0.01

No significant disparities were observed in the VDs of SCP, DCP, CC, and ChdV between the EaLM and MM groups across all nine 4 × 4 mm regions. However, the HM group exhibited a significant decrease in SCP VD in the ST (*p* = 0.030, Fig. 3A), S (*p* = 0.002, Fig. 3A), C (*p* = 0.017, Fig. 3A), I (*p* = 0.022, Fig. 3A) and IN regions (*p* = 0.027, Fig. 3A), DCP VD in ST (*p* = 0.006, Fig. 3B), S (*p* = 0.000, Fig. 3B), T (*p* = 0.013, Fig. 3B), C (*p* = 0.014, Fig. 3B), and I regions (*p* = 0.006, Fig. 3B), as well as ChdV VD in T (*p* = 0.039, Fig. 3D), N (*p* = 0.034, Fig. 3D), I region (*p* = 0.038, Fig. 3D), and IN regions (*p* = 0.033, Fig. 3D) when compared to the EaLM group. Additionally, SCP VD in I (*p* = 0.003, Fig. 3A), IN (*p* = 0.023, Fig. 3A) regions and DCP VD in I region (*p* = 0.005, Fig. 3B) displayed reductions in the HM group when compared to the MM group. Detailed information is provided in Additional file 2.

Surprisingly, compared to the MM group, the HM group exhibited significant increases in CC VD in ST (*p* = 0.019, Fig. 3C), S (*p* = 0.000, Fig. 3C), T (*p* = 0.025, Fig. 3C), C (*p* = 0.014, Fig. 3C), and N regions (*p* = 0.003, Fig. 3C). For additional information, please refer to Additional file 2.

Further correlation analysis revealed negative correlations between SCP VD in ST, S, C, I and IN regions with AL and SE. DCP VD in ST, S, SN, T, C, I and IN regions exhibited negative correlations with AL and/or SE. ChdV VD in ST, T, N, I, and IN regions were negatively correlated with AL and SE. Notably, CC VD in N region displayed positive correlations with AL and SE. (all: *p* < 0.05; Table 3).

Observing only the MM and HM groups, it was evident that CC VD in ST, S, SN, T, C and N regions showed positive correlations with both AL and SE. (all: *p* < 0.05; Additional file 3).

**Fig. 4:**
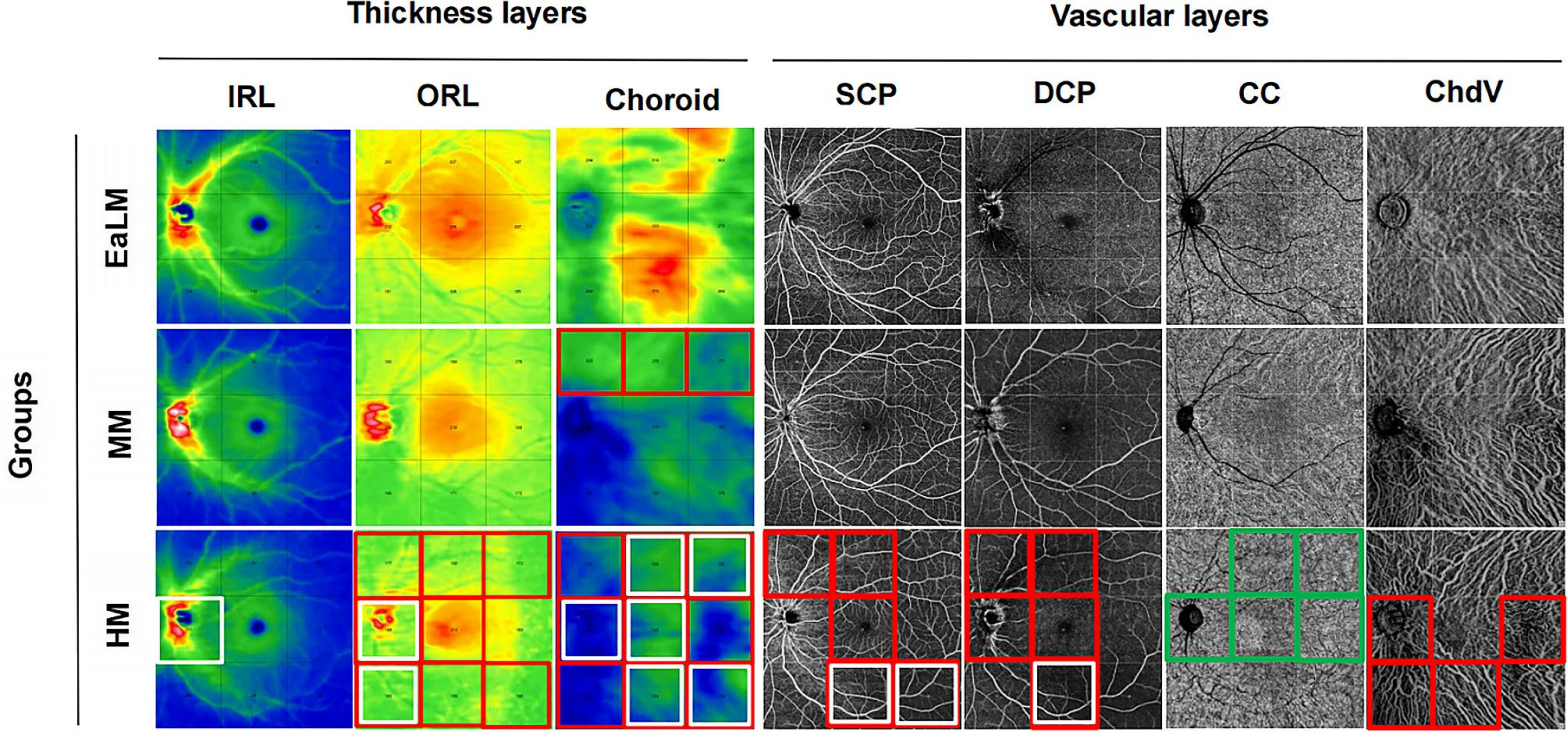
Summary of thickness (µm) and VD (%) differences in 9 regions of each layer of retina and choroid. Red boxes: decrease in thickness/VD of MM/HM compared to the EaLM group; White boxes: decrease in thickness/VD of HM compared to MM group; Green boxes: increase in VD of HM compared to MM. EaLM, Emmetropia and Low Myopia; MM, Moderate Myopia; HM, High Myopia. IRL, inner retinal layer; ORL, outer retinal layer; VD, vascular density; SCP, superficial capillary plexus; DCP, deep capillary plexus; CC, choriocapillaris; ChdV, choroid vessels

A summary of the thickness and VD differences in all nine regions of each retinal and choroidal layer is illustrated in Fig. 4.

**Table 3:** Correlation of VD (%) with AL (mm) and SE (-D) in 9 regions

Region	Layer	AL (mm)		SE (-D)	
		Coefficient	*P*-value	Coefficient	*P*-value
SCP	ST	-0.096	0.287	-0.191	0.033*
	S	-0.207	0.020*	-0.272	0.002**
	SN	-0.123	0.170	-0.163	0.068
	T	-0.122	0.174	-0.140	0.118
	C	-0.240	0.007**	-0.213	0.017*
	*N*	-0.057	0.527	0.024	0.788
	IT	-0.109	0.231	-0.093	0.306
	I	-0.179	0.047*	-0.229	0.011*
	IN	-0.210	0.019*	-0.250	0.005**
DCP	ST	-0.121	0.177	-0.243	0.006**
	S	-0.218	0.014*	-0.295	0.001**
	SN	-0.007	0.939	-0.046	0.610
	T	-0.191	0.032*	-0.242	0.006**
	C	-0.216	0.015*	-0.224	0.012*
	*N*	0.034	0.702	0.008	0.927
	IT	-0.097	0.286	-0.094	0.300
	I	-0.208	0.020*	-0.275	0.002**
	IN	-0.111	0.219	-0.191	0.033*
CC	ST	0.094	0.298	0.036	0.690
	S	0.148	0.097	0.148	0.098
	SN	0.122	0.172	0.118	0.189
	T	0.109	0.223	0.090	0.318
	C	0.093	0.298	0.107	0.233
	*N*	0.186	0.037*	0.211	0.018*
	IT	0.054	0.551	0.072	0.426
	I	0.031	0.727	0.000	0.996
	IN	-0.034	0.705	-0.096	0.285
ChdV	ST	-0.213	0.017*	-0.209	0.019*
	S	-0.173	0.052	-0.119	0.186
	SN	-0.168	0.060	-0.139	0.121
	T	-0.211	0.018*	-0.220	0.013*
	C	-0.170	0.056	-0.113	0.208
	*N*	-0.224	0.012*	-0.245	0.006**
	IT	-0.133	0.141	-0.141	0.120
	I	-0.222	0.013*	-0.222	0.013*
	IN	-0.221	0.013*	-0.246	0.006**

Statistically significant values are shown with */**, *P*<0.05 is marked by *, *P*<0.01 is marked by **. ST, supra-temporal; T, temporal; IT, infra-temporal; S, superior; C, central macular area; I, inferior; SN, supra-nasal; *N*, nasal; IN, infra-nasal; VD, vascular density; SCP, superficial capillary plexus; DCP, deep capillary plexus; CC, choriocapillaries; ChdV, choroid vessels; AL, axial length; SE, spherical equivalent

## Discussion

Myopia is highly prevalent, particularly concentrated in East and Southeast Asia [11]. A significant portion of individuals with HM will progress to pathological myopia (PM), which has led many researchers to explore the onset, progression and underlying mechanisms of myopia over the recent decades [11]. Previous studies using OCTA have primarily focused on the posterior pole of the eye [12]. However, in the context of HM, the eyeball exhibits uneven dilation and elongation [13]. Therefore, it is crucial to investigate changes in the fundus across various regions. Existing studies have shown that reduced choroidal blood flow is strongly associated with the progression of myopia. Currently, our understanding of ChdV concerning myopia is still in its exploratory stage. Gaining insights into alterations in structure and vessels, particularly choroid vascular, within the myopic fundus could provide valuable insights into the mechanisms of myopia progression. Hence, a wide-ranging region (12 × 12 mm encompassing the central macula) of the fundus in various degrees of myopia was divided into nine regions to assess the thickness and VD of the inner retina, outer retina, and choroid. Additionally, correlations between these characteristics and AL and SE were investigated.

Thinning of the inner retina was observed exclusively in the vicinity of the optic disc as myopia advanced. Both the outer retina (except in the temporal areas) and the choroid experienced thinning. Furthermore, elongation of the AL and increased refraction contributed to a reduction in the thickness of the outer retina and all choroidal regions. Previous studies have shown that the main site of eye axial elongation is the posterior equator, whereas the horizontal and vertical diameters of the eye are less dilated [14]. Changes in retinal nerve fibre layer (RNFL) thickness and RPE distribution pattern are strongly correlated with the pattern of ocular elongation [15]. Similarly, the posterior pole and mid-peripheral IRT we observed did not change significantly in the early stages of high myopia. The process of structural remodeling within the HM eye commences with the retina responding to visual stimuli, starting with the sclera [16]. This cascade of events results in the elongation of the ocular axis and may be best described as an “outward-to-inward” progression [17]. Consequently, scleral remodeling is likely the initial step in these structural alterations, followed by the initial thinning of the eyeball observed in the choroid and outer retina, which are contiguous with the sclera.

Our assessment encompassed not only the evaluation of retinal VD but also the quantification of ChdV VD, thus providing additional data to support potential future non-invasive vascular observations in the deeper layers of the fundus. VDs of both the SCP and DCP exhibited reductions across various regions. Previous research has consistently reported a decrease in blood flow density within the SCP and DCP in the macular region among individuals with HM [18]. However, some studies have noted elevated DCP VD in HM eyes within a 3 × 3 mm range [19], while others observed no changes in SCP and DCP in the macular region [20]. These discrepancies in study outcomes could be attributed to variations in the age of participants or the OCTA devices utilized. Reduced choroidal blood flow has been associated with increased axial length (AL), which may be an indication of myopia progression [21]. Our findings indicated a decrease in ChdV VD in HM eyes, aligning with prior reports.

When comparing Low and Moderate Myopia with HM, the outer retina and choroid exhibited extensive thinning in the high myopic eye. While, blood flow within the retina and choroid reduced in a few areas, implying that the body’s regulatory mechanisms for blood flow might be more responsive and efficient than those for structural changes as the eye progresses into high myopia. It is widely acknowledged that the growth of the eye under visual guidance is regulated by a sequential cascade of local chemical events involving signaling molecules. These signaling molecules, initiated by the retina, transmit information from the innermost to the outermost layers of the eye [22]. Subsequently, the extracellular matrix (ECM) within the sclera undergoes remodeling, resulting in alterations to the eye’s structure [23]. Ocular blood flow is subject to robust autoregulatory mechanisms for retinal blood flow and exogenous autonomic control over choroidal blood flow [24]. Consequently, autonomic control and self-regulation of blood flow may prove to be more efficient and sensitive than the regulation of eye structure through a cascade of chemical molecules.

Interestingly, we observed an increase in CC VD in HM eyes when compared to MM eyes in certain areas, demonstrating a fluctuating pattern of decline followed by an increase across all regions. The Choriocapillaris forms a dense capillary network that supplies oxygen and nutrients to the outer layers of the retina, including the RPE and photoreceptors [25]. Therefore, during the early stages of high myopia, it is plausible that there is a compensatory increase in CC VD in response to ischemia and hypoxia in deep retinal structures, such as the RPE. Further exploration of the molecular mechanisms could offer additional validation for this hypothesis. However, prior studies have reported no change or a decrease in CC in HM eyes [26, 27]. The compensatory function of CC VD may become impaired as myopia progresses to ultrahigh or pathological stages, further diminishing as blood flow to the choroidal vascular system decreases. Moreover, one study noted an elongation of the eye axis and a reduction in the density of retinal photoreceptors and RPE cells [28]. During this stage, an excessively high level of CC VD may no longer be necessary for compensation. Hence, there may be a point in the progression to HM where CC VD increases, subsequently decreasing.

When comparing eyes with EaLM to those with MM, only a reduction in CT in the upper three regions was evident. Conversely, eyes with HM displayed diverse levels of alteration in both the retinal and choroidal structure, alongside blood flow. This suggests that variations in upper choroidal thickness could serve as a sensitive indicator for the early detection of myopia development. It is imperative to allocate greater attention to this aspect in clinical practice. Furthermore, a recent study found that CVI was not significantly affected by high myopia but demonstrated a noticeable decrease in PM. Consequently, monitoring CVI could facilitate the early prediction of the onset of pathological myopia [29]. Nonetheless, significant changes in both fundus structure and blood flow have been observed in cases of HM. Therefore, it is crucial to emphasize early detection and the prevention of myopia progression. Additionally, although the correlation analysis yielded meaningful results, the low correlation coefficient suggests that axial elongation in myopia may be related not only to changes in fundus structure and blood flow but also to various other factors, necessitating further research into the underlying molecular mechanisms.

The prevalence of lattice degeneration (LD) increases progressively from emmetropia to moderate myopia and rises with increasing AL [30]. This suggests that myopia has begun to affect retinal structure and function after the onset of myopia. For this reason, it is extremely important to monitor the fundus on a regular basis once myopia has been diagnosed. This study offers fresh insights into comprehending early fundus lesions induced by myopia progression. What’s more, region-specific investigations may offer precise localization of fundus alterations in myopic eyes, potentially yielding new approaches for early detection of myopic lesions and understanding of the pathophysiological mechanism about myopia progression.

This study has certain limitations. For instance, the sample size is relatively small, and the research was conducted at a single center. Additionally, the study has a cross-sectional design, which restricts the ability to monitor dynamic changes in vascular density and structural thickness within the myopic fundus, making a long-term follow-up study necessary. Furthermore, although the mean value of the removed disc area was quantified when counting the data for the nasal region, there are some specific peripapillary disc border tissues in high myopia, such as the gamma and delta areas, which may introduce some error in the results.

In conclusion, cutting-edge ultra-widefield SS-OCTA shows that significant changes in retinal and choroid structure and vascular density occur as moderate myopia advances to high myopia. Efforts to curb myopia progression to this stage are essential, as the failure to do so may lead to the development of corresponding retinopathy.

### Electronic supplementary material

Below is the link to the electronic supplementary material.

Supplementary Material 1

Supplementary Material 2

Supplementary Material 3

## Data Availability

No datasets were generated or analysed during the current study.

## References

[1] Holden BA, Fricke TR, Wilson DA, Jong M, Naidoo KS, Sankaridurg P, Wong TY, Naduvilath TJ, Resnikoff S (2016). Global prevalence of myopia and high myopia and temporal trends from 2000 through 2050. Ophthalmology.

[2] Zheng F, Chua J, Ke M, Tan B, Yu M, Hu Q, Cheung CMG, Ang M, Lee SY, Wong TY, Schmetterer L, Wong CW, Hoang QV (2022). Quantitative OCT angiography of the retinal microvasculature and choriocapillaris in highly myopic eyes with myopic macular degeneration. Br J Ophthalmol.

[3] Zhang W, Li C, Gong Y, Liu N, Cao Y, Li Z, Zhang Y (2022). Advanced ultrawide-field optical coherence tomography angiography identifies previously undetectable changes in biomechanics-related parameters in nonpathological myopic fundus. Front Bioeng Biotechnol.

[4] Zhang S, Zhang G, Zhou X, Xu R, Wang S, Guan Z, Lu J, Srinivasalu N, Shen M, Jin Z, Qu J, Zhou X (2019). Changes in Choroidal Thickness and Choroidal Blood Perfusion in Guinea Pig Myopia. Invest Ophthalmol Vis Sci.

[5] Yang YS, Koh JW (2015). Choroidal Blood Flow change in eyes with high myopia. Korean J Ophthalmol.

[6] Nickla DL, Wallman J (2010). The multifunctional choroid. Prog Retin Eye Res.

[7] Liu F, Niu L, Guo J, Jian W, Shang J, Zhao J, Xue K, Zhou X (2023). Quantitative evaluation of retinal and choroidal vascularity and retrobulbar blood flow in patients with myopic anisometropia by CDI and OCTA. Br J Ophthalmol.

[8] Gao J, Rao CH, Li F, Liu L, Liu KJ (2022). Ultra-widefield swept-source Optical Coherence Tomography Angiography in the Assessment of Choroidal Changes in Young adults with myopia. Transl Vis Sci Technol.

[9] Zeng Q, Yao Y, Li S, Yang Z, Qu J, Zhao M (2022). Comparison of swept-source OCTA and indocyanine green angiography in central serous chorioretinopathy. BMC Ophthalmol.

[10] Qi Z, Si Y, Feng F, Zhu J, Yang X, Wang W, Zhang Y, Cui Y (2023). Analysis of retinal and choroidal characteristics in patients with early diabetic retinopathy using WSS-OCTA. Front Endocrinol (Lausanne).

[11] Morgan IG, French AN, Ashby RS, Guo X, Ding X, He M, Rose KA (2018). The epidemics of myopia: Aetiology and prevention. Prog Retin Eye Res.

[12] Liu M, Wang P, Hu X, Zhu C, Yuan Y, Ke B (2021). Myopia-related stepwise and quadrant retinal microvascular alteration and its correlation with axial length. Eye (Lond).

[13] Moon JY, Garg I, Cui Y, Katz R, Zhu Y, Le R, Lu Y, Lu ES, Ludwig CA, Elze T, Wu DM, Eliott D, Miller JW, Kim LA, Husain D, Vavvas DG, Miller JB (2023). Wide-field swept-source optical coherence tomography angiography in the assessment of retinal microvasculature and choroidal thickness in patients with myopia. Br J Ophthalmol.

[14] Matsumura S, Kuo AN, Saw SM (2019). An update of Eye shape and myopia. Eye Contact Lens.

[15] Jonas JB, Ohno-Matsui K, Holbach L, Panda-Jonas S (2017). Retinal pigment epithelium cell density in relationship to axial length in human eyes. Acta Ophthalmol.

[16] Boote C, Sigal IA, Grytz R, Hua Y, Nguyen TD, Girard MJA (2020). Scleral structure and biomechanics. Prog Retin Eye Res.

[17] Lim LS, Yang X, Gazzard G, Lin X, Sng C, Saw SM, Qiu A (2011). Variations in eye volume, surface area, and shape with refractive error in young children by magnetic resonance imaging analysis. Invest Ophthalmol Vis Sci.

[18] Li M, Yang Y, Jiang H, Gregori G, Roisman L, Zheng F, Ke B, Qu D, Wang J (2017). Retinal Microvascular Network and Microcirculation assessments in high myopia. Am J Ophthalmol.

[19] Milani P, Montesano G, Rossetti L, Bergamini F, Pece A (2018). Vessel density, retinal thickness, and choriocapillaris vascular flow in myopic eyes on OCT angiography. Graefes Arch Clin Exp Ophthalmol.

[20] Mo J, Duan A, Chan S, Wang X, Wei W. Vascular flow density in pathological myopia: an optical coherence tomography angiography study. BMJ Open 2017, 7 (2), e013571. 10.1136/bmjopen-2016-013571 PMC5294002 28159853

[21] Meng W, Butterworth J, Malecaze F, Calvas P (2011). Axial length of myopia: a review of current research. Ophthalmologica.

[22] Rada JA, Shelton S, Norton TT (2006). The sclera and myopia. Exp Eye Res.

[23] Summers JA (2013). The choroid as a sclera growth regulator. Exp Eye Res.

[24] McDougal DH, Gamlin PD (2015). Autonomic control of the eye. Compr Physiol.

[25] Reiner A, Fitzgerald MEC, Del Mar N, Li C (2018). Neural control of choroidal blood flow. Prog Retin Eye Res.

[26] Liu X, Lin Z, Wang F, Peng X, He W, Chen D, Shen M, Lu F, Jiang J (2021). Choroidal thickness and choriocapillaris vascular density in myopic anisometropia. Eye Vis (Lond).

[27] Xu A, Sun G, Duan C, Chen Z, Chen C. Quantitative Assessment of three-Dimensional Choroidal Vascularity and Choriocapillaris Flow Signal Voids in myopic patients using SS-OCTA. Diagnostics (Basel) 2021, *11* (11). 10.3390/diagnostics11111948 PMC8618547 34829297

[28] Panda-Jonas S, Jonas JB, Jonas RA (2022). Photoreceptor density in relation to axial length and retinal location in human eyes. Sci Rep.

[29] Wang Y, Chen S, Lin J, Chen W, Huang H, Fan X, Cao X, Shen M, Ye J, Zhu S, Xue A, Lu F, Shao Y (2022). Vascular changes of the Choroid and their correlations with visual acuity in pathological myopia. Invest Ophthalmol Vis Sci.

[30] Celorio JM, Pruett RC (1991). Prevalence of lattice degeneration and its relation to axial length in severe myopia. Am J Ophthalmol.

