# Supplementary material for: Observation of structural and vascular features of retina and choroid in myopia using ultra-widefield SS-OCTA
Source: BMC Ophthalmol. 2024 May 7;24:208. doi: 10.1186/s12886-024-03473-y (PMC11075211; doi:10.1186/s12886-024-03473-y)
Supplement: Supplementary file 1 — Supplementary Material 1 [file 12886_2024_3473_MOESM1_ESM.docx]

**Table S1. Thickness (μm) of retina and choroid in 9 regions**

| Layer | Region | EaLM | MM | HM | P | P_1_ | P_2_ | P_3_ |
| --- | --- | --- | --- | --- | --- | --- | --- | --- |
| IRT | ST | 73.76±2.75 | 71.39±2.31 | 74.02±2.87 | 0.768 | 1.000 | 1.000 | 1.000 |
|  | S | 108.33±2.70 | 104.10±2.12 | 104.89±2.54 | 0.511 | 0.828 | 1.000 | 1.000 |
|  | SN | 118.38±3.12 | 115.21±2.92 | 112.80±3.09 | 0.464 | 1.000 | 0.652 | 1.000 |
|  | T | 86.85±4.66 | 79.15±2.76 | 83.44±3.49 | 0.387 | 0.517 | 1.000 | 1.000 |
|  | C | 117.48±1.69 | 115.18±1.66 | 114.59±1.91 | 0.536 | 1.000 | 0.823 | 1.000 |
|  | N | 148.70±4.76 | 159.79±3.83 | 142.87±3.08 | 0.005** | 0.169 | 0.843 | 0.004** |
|  | IT | 73.84±3.15 | 70.92±2.32 | 72.96±3.08 | 0.804 | 1.000 | 1.000 | 1.000 |
|  | I | 100.58±3.15 | 95.87±2.57 | 95.89±2.95 | 0.484 | 0.911 | 0.810 | 1.000 |
|  | IN | 99.25±5.01 | 93.42±4.25 | 96.17±4.70 | 0.731 | 1.000 | 1.000 | 1.000 |
|  |  |  |  |  |  |  |  |  |
| ORT | ST | 194.64±1.86 | 192.35±1.62 | 189.06±1.34 | 0.040* | 1.000 | 0.041* | 0.384 |
|  | S | 202.58±2.36 | 197.50±1.98 | 194.67±1.78 | 0.025* | 0.308 | 0.020* | 0.913 |
|  | SN | 201.52±1.93 | 195.39±1.95 | 192.13±1.53 | 0.001** | 0.079 | 0.001** | 0.540 |
|  | T | 201.85±2.57 | 197.24±1.72 | 195.26±1.83 | 0.076 | 0.419 | 0.072 | 1.000 |
|  | C | 226.15±2.11 | 223.45±1.59 | 218.54±1.74 | 0.011* | 1.000 | 0.012* | 0.153 |
|  | N | 210.61±2.38 | 210.95±2.62 | 202.31±1.87 | 0.007* | 1.000 | 0.032* | 0.017* |
|  | IT | 190.19±3.96 | 181.72±1.53 | 178.41±1.61 | 0.002** | 0.060 | 0.002** | 0.896 |
|  | I | 187.55±3.03 | 183.76±2.20 | 179.09±2.10 | 0.044* | 0.920 | 0.043* | 0.476 |
|  | IN | 185.66±2.25 | 183.76±2.36 | 175.85±1.51 | 0.001** | 1.000 | 0.002** | 0.012* |
|  |  |  |  |  |  |  |  |  |
| CT | ST | 290.00±11.78 | 253.74±9.71 | 225.94±8.36 | 0.000** | 0.048* | 0.000** | 0.115 |
|  | S | 314.03±10.96 | 264.62±11.73 | 222.13±8.79 | 0.000** | 0.007** | 0.000** | 0.009** |
|  | SN | 268.39±13.66 | 226.26±11.02 | 180.13±7.76 | 0.000** | 0.026* | 0.000** | 0.004** |
|  | T | 285.55±11.68 | 265.56±10.85 | 211.37±8.15 | 0.000** | 0.574 | 0.000** | 0.000** |
|  | C | 287.94±11.33 | 252.26±12.56 | 193.89±7.88 | 0.000** | 0.076 | 0.000** | 0.000** |
|  | N | 175.82±10.36 | 156.13±10.55 | 126.48±7.06 | 0.001** | 0.471 | 0.001** | 0.052 |
|  | IT | 250.19±11.91 | 238.81±12.50 | 197.44±7.95 | 0.001** | 1.000 | 0.002** | 0.013* |
|  | I | 263.36±12.43 | 234.47±14.08 | 189.65±8.05 | 0.000** | 0.279 | 0.000** | 0.011* |
|  | IN | 184.48±11.11 | 167.24±11.78 | 139.04±6.55 | 0.003** | 0.702 | 0.003** | 0.090 |

Statistically significant values are shown with */**, p＜0.05 is marked by *, p＜0.01 is marked by **. EaLM, Emmetropia and Low Myopia; MM, Moderate Myopia; HM, High Myopia. IRT, inner retinal thickness; ORT, outer retinal thickness; CT, choroid thickness. ST, supra-temporal (A); S, superior (B); SN, supra-nasal (C); T, temporal (D); C, central macular (E); N, nasal (F); IT, infra-temporal (G); I, inferior (H); IN, and infra-nasal (I). P: difference between 3 groups by ANOVA; P_1_: EaLM vs. MM; P_2_: EaLM vs. HM; P_3_: MM vs. HM.
