# Supplementary material for: Observation of structural and vascular features of retina and choroid in myopia using ultra-widefield SS-OCTA
Source: BMC Ophthalmol. 2024 May 7;24:208. doi: 10.1186/s12886-024-03473-y (PMC11075211; doi:10.1186/s12886-024-03473-y)
Supplement: Supplementary file 2 — Supplementary Material 2 [file 12886_2024_3473_MOESM2_ESM.docx]

**Table S2. VD (%) of retina and choroid in 9 regions**

| Layer | Region | EaLM | MM | HM | P | P_1_ | P_2_ | P_3_ |
| --- | --- | --- | --- | --- | --- | --- | --- | --- |
| SCP | ST | 37.76±1.43 | 33.55±1.74 | 32.65±1.04 | 0.030* | 0.142 | 0.030* | 1.000 |
|  | S | 44.24±0.83 | 41.64±0.93 | 40.06±0.74 | 0.003** | 0.131 | 0.002** | 0.493 |
|  | SN | 44.09±0.94 | 43.46±0.80 | 42.02±0.77 | 0.184 | 1.000 | 0.252 | 0.614 |
|  | T | 37.97±1.37 | 36.54±1.21 | 35.00±0.94 | 0.187 | 1.000 | 0.213 | 0.969 |
|  | C | 41.97±0.97 | 40.38±1.00 | 38.50±0.69 | 0.019* | 0.698 | 0.017* | 0.333 |
|  | N | 48.48±0.68 | 48.87±0.52 | 48.67±0.50 | 0.901 | 1.000 | 1.000 | 1.000 |
|  | IT | 36.72±1.54 | 38.30±1.13 | 35.26±1.04 | 0.186 | 1.000 | 1.000 | 0.204 |
|  | I | 44.22±1.03 | 44.74±0.99 | 41.09±0.52 | 0.002** | 1.000 | 0.022* | 0.003** |
|  | IN | 41.24±1.15 | 41.16±1.00 | 37.52±0.85 | 0.007* | 1.000 | 0.027* | 0.023* |
|  |  |  |  |  |  |  |  |  |
| DCP | ST | 40.97±1.24 | 35.74±1.94 | 34.31±1.22 | 0.007** | 0.070 | 0.006** | 1.000 |
|  | S | 43.97±0.91 | 40.38±1.17 | 38.02±1.02 | 0.001** | 0.091 | 0.000** | 0.318 |
|  | SN | 38.42±1.45 | 38.79±1.13 | 37.52±1.05 | 0.713 | 1.000 | 1.000 | 1.000 |
|  | T | 41.91±1.18 | 39.26±1.23 | 37.02±1.08 | 0.016* | 0.425 | 0.013* | 0.488 |
|  | C | 46.15±1.35 | 43.21±1.64 | 40.33±1.24 | 0.017** | 0.528 | 0.014* | 0.414 |
|  | N | 41.61±0.99 | 41.49±1.11 | 41.74±0.92 | 0.983 | 1.000 | 1.000 | 1.000 |
|  | IT | 40.03±1.61 | 41.54±1.11 | 38.81±1.07 | 0.275 | 1.000 | 1.000 | 0.327 |
|  | I | 46.00±1.05 | 45.87±1.43 | 41.37±0.66 | 0.001** | 1.000 | 0.006** | 0.005** |
|  | IN | 41.82±1.18 | 41.74±1.21 | 38.67±1.01 | 0.065 | 1.000 | 0.156 | 0.145 |
|  |  |  |  |  |  |  |  |  |
| CC | ST | 55.97±1.34 | 51.13±1.51 | 56.09±1.12 | 0.013* | 0.053 | 1.000 | 0.019* |
|  | S | 55.67±1.09 | 52.21±0.90 | 57.56±0.91 | 0.000** | 0.063 | 0.523 | 0.000** |
|  | SN | 55.70±1.33 | 54.44±1.03 | 57.50±0.91 | 0.103 | 1.000 | 0.715 | 0.109 |
|  | T | 57.48±1.35 | 54.72±1.31 | 59.30±1.12 | 0.030* | 0.455 | 0.942 | 0.025* |
|  | C | 55.58±1.25 | 52.85±1.28 | 57.39±0.99 | 0.017* | 0.376 | 0.823 | 0.014* |
|  | N | 55.76±0.97 | 54.28±0.89 | 58.17±0.72 | 0.003** | 0.761 | 0.141 | 0.003** |
|  | IT | 56.66±1.83 | 56.57±1.45 | 58.70±1.15 | 0.448 | 1.000 | 0.940 | 0.816 |
|  | I | 59.39±1.44 | 57.74±1.46 | 59.31±1.01 | 0.599 | 1.000 | 1.000 | 1.000 |
|  | IN | 59.82±1.49 | 57.05±1.21 | 58.00±1.09 | 0.342 | 0.446 | 0.917 | 1.000 |
|  |  |  |  |  |  |  |  |  |
| ChdV | ST | 56.12±0.88 | 54.68±0.94 | 53.52±0.62 | 0.071 | 0.712 | 0.067 | 0.844 |
|  | S | 55.30±0.92 | 54.00±0.97 | 53.72±0.66 | 0.393 | 0.917 | 0.533 | 1.000 |
|  | SN | 55.64±0.92 | 54.44±0.95 | 54.17±0.51 | 0.387 | 0.915 | 0.539 | 1.000 |
|  | T | 55.91±0.88 | 54.64±0.83 | 53.19±0.62 | 0.041* | 0.828 | 0.039* | 0.479 |
|  | C | 55.33±0.91 | 54.18±0.84 | 53.98±0.57 | 0.424 | 0.941 | 0.620 | 1.000 |
|  | N | 54.61±0.76 | 52.33±0.96 | 51.80±0.60 | 0.034* | 0.163 | 0.034* | 1.000 |
|  | IT | 56.09±1.06 | 55.08±1.15 | 54.35±0.64 | 0.406 | 1.000 | 0.542 | 1.000 |
|  | I | 56.64±0.82 | 54.76±1.02 | 53.74±0.62 | 0.044* | 0.391 | 0.038* | 1.000 |
|  | IN | 56.67±0.73 | 54.79±1.16 | 53.26±0.81 | 0.038* | 0.566 | 0.033* | 0.684 |

Statistically significant values are shown with */**, p＜0.05 is marked by *, p＜0.01 is marked by **. EaLM, Emmetropia and Low Myopia; MM, Moderate Myopia; HM, High Myopia. VD, vascular density; SCP, superficial capillary plexus; DCP, deep capillary plexus; CC, choriocapillaries; ChdV, choroid vessels. ST, supra-temporal (A); S, superior (B); SN, supra-nasal (C); T, temporal (D); C, central macular (E); N, nasal (F); IT, infra-temporal (G); I, inferior (H); IN, and infra-nasal (I). P: difference between 3 groups by ANOVA; P_1_: EaLM vs. MM; P_2_: EaLM vs. HM; P_3_: MM vs. HM.
