# Supplementary material for: Observation of structural and vascular features of retina and choroid in myopia using ultra-widefield SS-OCTA
Source: BMC Ophthalmol. 2024 May 7;24:208. doi: 10.1186/s12886-024-03473-y (PMC11075211; doi:10.1186/s12886-024-03473-y)
Supplement: Supplementary file 3 — Supplementary Material 3 [file 12886_2024_3473_MOESM3_ESM.docx]

**Table S3. Correlation of CC VD (%) with AL (mm) and SE (-D) in 9 regions of MM and HM groups**

| Layer | Region | AL (mm) | | SE (-D) | |
| --- | --- | --- | --- | --- | --- |
|  |  | Coefficient | P | Coefficient | P |
| CC | ST | 0.282 | 0.006** | 0.284 | 0.006** |
|  | S | 0.342 | 0.001** | 0.400 | 0.000** |
|  | SN | 0.243 | 0.019* | 0.233 | 0.024* |
|  | T | 0.225 | 0.030* | 0.258 | 0.013* |
|  | C | 0.231 | 0.026* | 0.289 | 0.005** |
|  | N | 0.302 | 0.003** | 0.388 | 0.000** |
|  | IT | 0.127 | 0.231 | 0.140 | 0.186 |
|  | I | 0.149 | 0.155 | 0.132 | 0.209 |
|  | IN | 0.142 | 0.176 | 0.091 | 0.388 |

Statistically significant values are shown with */**, P＜0.05 is marked by *, P＜0.01 is marked by **. VD, vascular density; CC, choriocapillaries; ChdV, choroid vessels; AL, axial length; SE, spherical equivalent; MM, moderate myopia; HM, high myopia. ST, supra-temporal; T, temporal; IT, infra-temporal; S, superior; C, central macular area; I, inferior; SN, supra-nasal; N, nasal; IN, infra-nasal.
